# The M310T mutation in the GATA4 gene is a novel pathogenic target of the familial atrial septal defect

**DOI:** 10.1186/s12872-020-01822-5

**Published:** 2021-01-06

**Authors:** Haisong Bu, Guowen Sun, Yun Zhu, Yifeng Yang, Zhiping Tan, Tianli Zhao, Shijun Hu

**Affiliations:** 1 Department of Cardiovascular Surgery, The Second Xiangya Hospital, Central South University, 139 Renmin Central Road, Changsha, 410011 Hunan People’s Republic of China; 2 Department of Cardiothoracic Surgery, Chenzhou No. 1 People’s Hospital, Chenzhou, 423000 Hunan People’s Republic of China; 3 Central South University Center for Clinical Gene Diagnosis and Treatment, the Second Xiangya Hospital, Central South University, Changsha, 410011 Hunan People’s Republic of China; 4 Department of Cardiovascular Surgery, The German Heart Centre, 80636 Munich, Germany; 5 Department of Biochemistry and Molecular Biology, Mayo Clinic, Rochester, MN 55902 USA

**Keywords:** Atrial septal defect, *GATA4* gene, Whole-exome sequencing, Nuclear localization signal, Arrhythmia

## Abstract

**Background:**

Although most cases of atrial septal defect (ASD) are sporadic, familial cases have been reported, which may be caused by mutation of transcription factor GATA binding protein 4 (*GATA4*). Herein we combined whole-exome sequencing and bioinformatics strategies to identify a novel mutation in *GATA4* accounting for the etiology in a Chinese family with ASD.

**Methods:**

We identified kindred spanning 3 generations in which 3 of 12 (25.0%) individuals had ASD. Punctilious records for the subjects included complete physical examination, transthoracic echocardiography, electrocardiograph and surgical confirming. Whole-exome capture and high-throughput sequencing were performed on the proband III.1. Sanger sequencing was used to validate the candidate variants, and segregation analyses were performed in the family members.

**Results:**

Direct sequencing of *GATA4* from the genomic DNA of family members identified a T-to-C transition at nucleotide 929 in exon 5 that predicted a methionine to threonine substitution at codon 310 (M310T) in the nuclear localization signal (NLS) region. Two affected members (II.2 and III.3) and the proband (III.1) who was recognized as a carrier exhibited this mutation, whereas the other unaffected family members or control individuals did not. More importantly, the mutation *GATA4* (c.T929C: p.M310T) has not been reported previously in either familial or sporadic cases of congenital heart defects (CHD).

**Conclusions:**

We identified for the first time a novel M310T mutation in the *GATA4* gene that is located in the NLS region and leads to family ASD with arrhythmias. However, the mechanism by which this pathogenic mutation contributes to the development of heart defect and tachyarrhythmias remains to be ascertained.

## Introduction

Atrial septal defect (ASD) is a common cardiovascular malformation, accounting for 10% of congenital heart defects (CHD), which is one of the major birth defects in the world. ASD is often reported in sporadic form; however, the reported familial cases have more research value [1–3]. ASD may be isolated or associated with other CHDs, such as pulmonary valve stenosis (PVS), ventricular septal defect (VSD) and conduction defects, one of the study found that GATA4 genetic variations are associated with ASD, TOF and VSD in South Indian patients. In silico analysis provides further evidence that some of the observed mutations are pathogenic [4]. In addition, persistent left to right blood shunt may result in atrial dysfunctions and atrial arrhythmias, in the absence of surgical or catheter-based repair [5]. Therefore, CHD is still a serious threat to human, so the early prenatal screening and diagnosis for this type of birth defect are urgently required.

Although the CHD etiology is too complicated to be well characterized because of the complexity of heart development, numerous intrinsic factors [6] (genetic factors) and extrinsic threats [7] (environmental) were identified as contribution to CHD. Many candidate genes such as *GATA4*, *TBX5*, *NKX2.5*, *BMP4* and *HAND1* have been proven to be responsible for heart development and diseases [8]. *GATA4* is one of the most widely investigated genes in CHD, with over 100 known mutation sites, which are related to the structural heart defect such as ASD, VSD, and PVS [9]. Recently, the phenotypic genetics of familial ASD has been widely investigated, and transcription factors as an important mediator in cardiac development are still the focus of attention [10–12]. Intrinsic factors have been identified as a major contributor to the pathogenesis of family ASD with the development of sequencing technology [13], and mutations in the *GATA4* gene have been identified as a pathogenic factor of familial ASD [3, 14].

The zinc-finger transcription factors were encoded by GATA binding protein 4 (*GATA4*), which is essential for heart development [15, 16] and considered to be a gene regulating embryogenesis and myocardial differentiation and function, and bound the GATA motif which is present in the promoters of many genes [17]. *GATA4* has 442 amino acids, including the N-terminus zinc fingers (NZf), the C-terminus zinc fingers (CZf) and the nuclear localization signal (NLS) [2]. More importantly, the 271–322 amino acid fragment in the DNA binding domain has been reported and proved to be the smallest functional NLS region, which is vital to the process of cardiac development [18]. In the current study, we checked out a clinically characterized family with a diagnosis of ASD. We found an obvious autosomal-dominant inheritance with reduced penetrance in this family. In addition, after performing surgical confirming and surgical repairs on patients, we conducted a clinical and genetic analysis and identified for the first time a novel pathogenic mutation of *GATA4* in the NLS region (NM_002052: exon5: c.T929C: p.M310T) by whole-exome sequencing of the patient in the family, which was confirmed by Sanger sequencing. Taken together, our study strongly suggests that the dominant family ASD involved in this study may be caused by *GATA4* gene deficiency.

## Patients and methods

### Patients and clinical examination

**Fig. 1:**
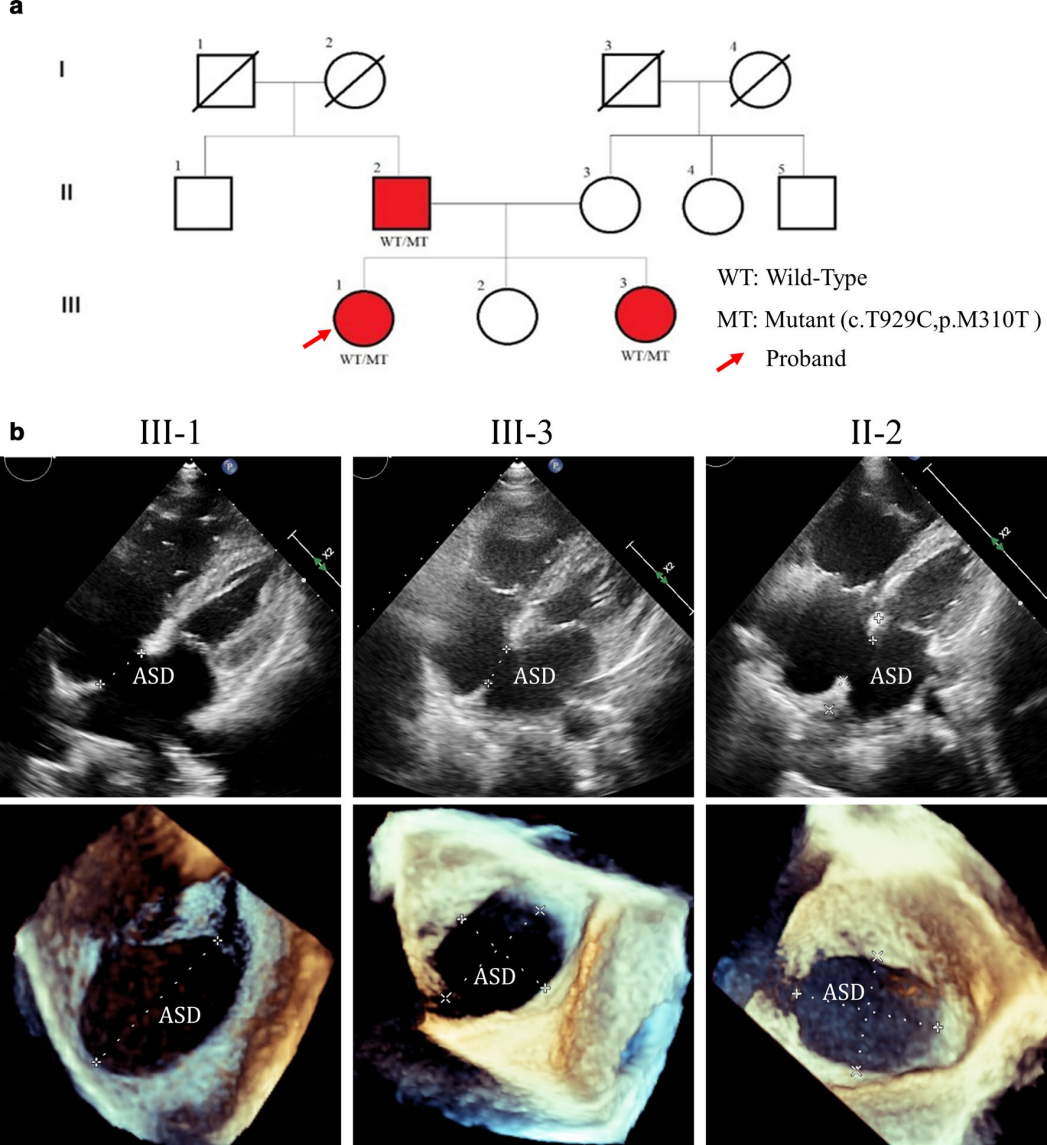
**a** Pedigree of the affected family is comprised of three generations. The squares and circles indicate males and females, respectively. Participating members of each generation are indicated numerically. The arrow appoints the proband of the family. The mutation, c.T929C in *GATA4*, has been demonstrated that segregated in this family; **b** All members with the heart defects were diagnosed by transthoracic echocardiography. ASD is clearly displayed through 3D reconstruction (bottom). ASD, atrial septal defect

The present study enrolled 3 patients (Fig. 1a) with ASD and arrhythmia from The Second Xiangya Hospital of Central South University (Changsha, China). The study group comprised of 5 male and 7 female patients (Table 1). Punctilious records for the subjects included a complete physical examination, a transthoracic echocardiography, a 12-lead electrocardiograph and a surgical confirming. All noninvasive exam results are confirmed in the surgery.

**Table 1:** Clinical features in members of family

Member	Gender	Age (year)	Structural diagnosis	ECG diagnosis	TTE feature				Mutation	Surgery required
					RA (mm)	RV (mm)	LVEF (%)	AO (mm)		
III1*	Female	14	Secundum ASD with ASA, PAVSD, PS, and PLSVC	Sinus tachycardia, frequent junctional premature beat with aberrant ventricular conduction	50	37	60	24	WT/p. M310T	+
III2	Female	8	N/A	IRBBB	22	33	69	17	WT/ WT	–
III3	Female	4	Secundum ASD, mild PS and PLSVC	Sinus arrhythmia (sinus tachycardia alternating with sinus bradycardia), cardiac rhythm migration between atrionector and junction	34	31	56	15	WT/p. M310T	+
II1	Male	48	N/A	N/A	43	40	67	25	WT/WT	–
II2	Male	44	Secundum ASD, PS and AAD	Atrial fibrillation paroxysmal ventricular tachycardia	71	37	59	37	WT/p. M310T	+
II3	Female	39	N/A	N/A	40	38	62	22	WT/WT	–
II4	Male	36	N/A	IRBBB	38	36	63	21	WT/WT	–
II5	Female	32	N/A	N/A	35	32	65	20	WT/WT	–
I1	Male	72	N/A	IRBBB	45	38	57	25	WT/WT	–
I2	Female	68	PFO	N/A	43	32	61	20	WT/WT	–
I3	Male	67	N/A	N/A	44	33	59	23	WT/WT	–
I4	Female	60	N/A	N/A	43	35	60	22	WT/WT	–

ECG, electrocardiograph; TTE, transthoracic echocardiography; AAD, ascending aortic dilatation; PAVSD, partial atrioventricular septal defect; ASD, atrial septal defect; PS, pulmonary stenosis; PLSVC, persistent left superior vena cava; ASA, atrial septal aneurysm; IRBBB, incomplete right bundle brunch block; RA, right atrium; RV, right ventricle; AO, aortic; LVEF, left ventricle ejection fraction

^*^Proband

The study protocol was approved by Review Board of the Second Xiangya Hospital of Central South University (Changsha, China). Written informed consents for the publication of the patient’s information were obtained from the parents of the patient and patents themselves.

### DNA extraction

Genomic DNA was extracted from peripheral blood lymphocytes of each patient and the family members. Genomic DNA was prepared for testing with a DNeasy Blood and Tissue kit (Qiagen, Valencia, CA, USA) on a QIA cube automated DNA-extraction robot (Qiagen, Hilden, Germany) [19]. The quality and quantity of the DNA samples were measured by a NanoDrop 2000 spectrophotometer (Thermo Fisher Scientific, Inc., Waltham, MA, USA), after which 2 µl DNA from each sample was used for analysis [20, 21].

### Whole-exome sequencing (WES) and filtering

**Fig. 2:**
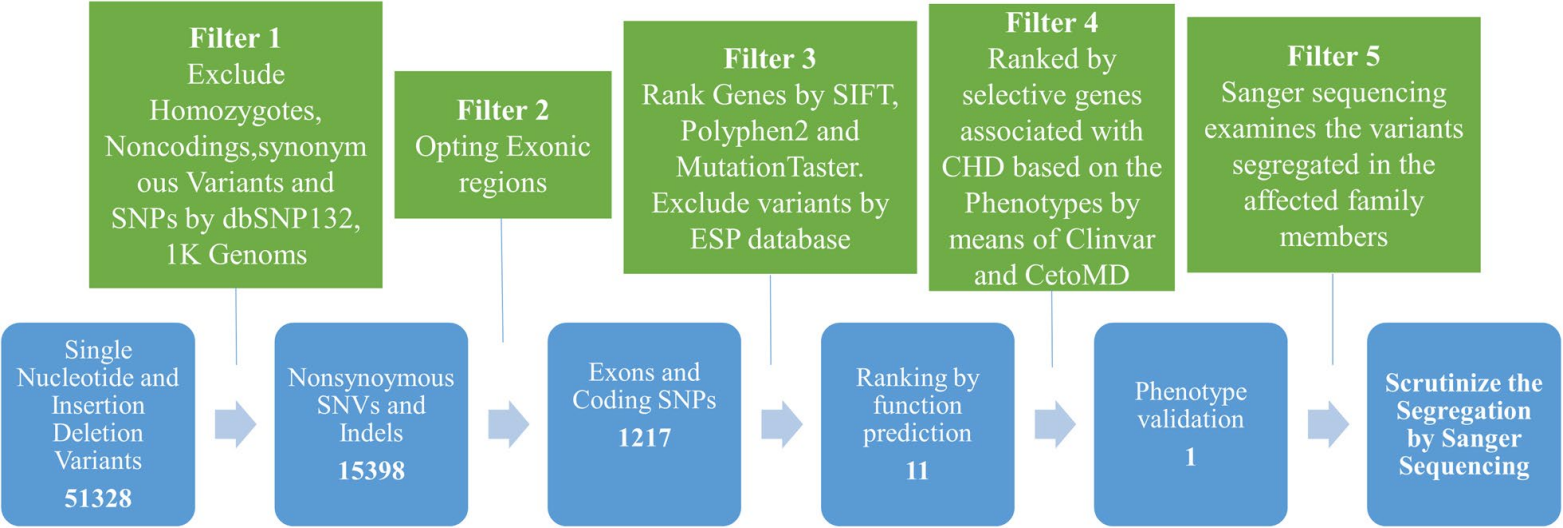
Schematic representation of filtering strategies applied in this research. The filtering process was applied according to several strategies that are demonstrated in the schematic representation

At the Novogene Bioinformatics Institute (Beijing, China), we used whole-exome capture and high-throughput sequencing (HTS) technology to detect the proband III.1 systematically. In short, the Agilent SureSelect Human All ExonV5 Kit (Agilent, California, USA) was purchased and used to capture the whole exomes, which were then sequenced on the Illumina HiSeq. 2500 platform [22]. Consistent with the human reference genome (UCSC hg19), the details of the sequencing data are shown in Fig. 2. The following criteria were used as filtering criteria for single nucleotide variants (SNVs) and short InDels [23]: (1) synonymous mutations and variants, such as intergenic variation, intronic variation, and UTR regions variation, should be excluded in subsequent analysis; (2) High-frequency (minor allele frequency > 0.01) polymorphisms should be excluded from the databases, such as 1000 Genomes Project, ExAC, ESP6500, and Novogene Bioinformatics Institute internal Exome Sequencing databases; (3) According to the identification principle of new pathogenic genes, the known pathogenic genes should be excluded, and then the variations of 42 known CHD-related genes [24] were listed. Any compound heterozygotes of known genes can be found by this method. (4) Runs of homozygosity (ROH) analysis [22], is a vital method because it can effectively eliminate false-positive variation in the situation of a large number of deletion on the other allele, should be performed due to the examination of consanguineous families. Refer to Fig. 2 for detailed filtering steps.

### Bioinformatics analysis

Bioinformatics programs, such as MutationTaster, Sift, PROVEAN, Polyphen-2, and LRT, were used to predict the effects of identified variants on protein function, and the Swiss model tools were used to determine the potential effects on protein structure. To further assess the protective effect of the identified variants, we obtained and compared the amino acid sequences of target genes in different species from MutationTaster (http://www.mutationtaster.org), and then the domain analyses were carried out in NCBI Conserved Domains websites (http://www.ncbi.nlm.nih.gov/Structure/cdd/wrpsb.cgi).

### Mutation validation and co-segregation analysis

Sanger sequencing in forward and reverse directions was used to validate the candidate variants identified by WES, and segregation analyses were performed in the family members. Primers pairs used to amplify fragments encompassing individual variants were designed using an online tool (Primer Quest, IDT) (http://www.idtdna.com/Primerquest/Home/Index), and the sequences of the primers as follows: Forward primer: 5′-TCTTTCTCGCTGAGTTCC-3′, Reverse primer: 5′-TTGAGTTGAGCCTGCTTC-3'.

## Results

### Clinical features

We identified 3 patients, the father and two of the daughters, with multiple complex phenotypes including CHD and arrhythmia in a Chinese family (Fig. 1a). The proband III.1, a 14-year-old patient from Hunan province of Central-South China, was diagnosed as secundum ASD with atrial septal aneurysm, partial atrioventricular septal defect (PAVSD), PVS and persistent left superior vena cava by transthoracic echocardiography (Fig. 1b) and sinus tachycardia, frequent junctional premature beat with aberrant ventricular conduction by HOLTER.

The father II.2 was diagnosed as secundum ASD, PS and ascending aortic dilatation by transthoracic echocardiography (Fig. 1b) and atrial fibrillation paroxysmal ventricular tachycardia by HOLTER. The other patient III.3 was diagnosed as secundum ASD, mild PS and persistent left superior vena cava by transthoracic echocardiography (Fig. 1b) and sinus arrhythmia (sinus tachycardia alternating with sinus bradycardia), cardiac rhythm migration between atrionector and junction by HOLTER. Other members of the family did not show any structural heart defect or severe arrhythmia in the examination. All structural defects were confirmed in the operation. Proband III.1 presented ventricular tachycardia after sternotomy and the heart was irritable in the whole procedure. All clinical details have been reported in Table 1.

### Genetic analysis

WES was performed on proband III.1 and the sequence read of 4.7Gbp was generated. The mean depth of the target region is 47.3 × and 95.7% of the targeted bases were covered sufficiently to pass the threshold for SNVs and InDels (Additional file 1: Table S1). Several filtering methods were performed to analyze the known SNVs and InDels. After alignment and SNV calling, 51,328 variants were detected in the proband’s exome. We used several databases to exclude all exonic InDels, non-synonymous variants, and nonsense and splice-site SNVs. Then we ranked genes by Sift, Polyphen-2, and Mutation Taster, 11 variants were identified and prioritized them by patients’ phenotype. 42 CHD-related genes were used to cross-contrast the 11 variants. Eventually, only the mutation *GATA4* NM_002052: exon5: c.T929C: p.M310T could be confirmed in the other two affected family members (II.2 III.3) and could not be detected in other healthy parent or normal control.

**Fig. 3:**
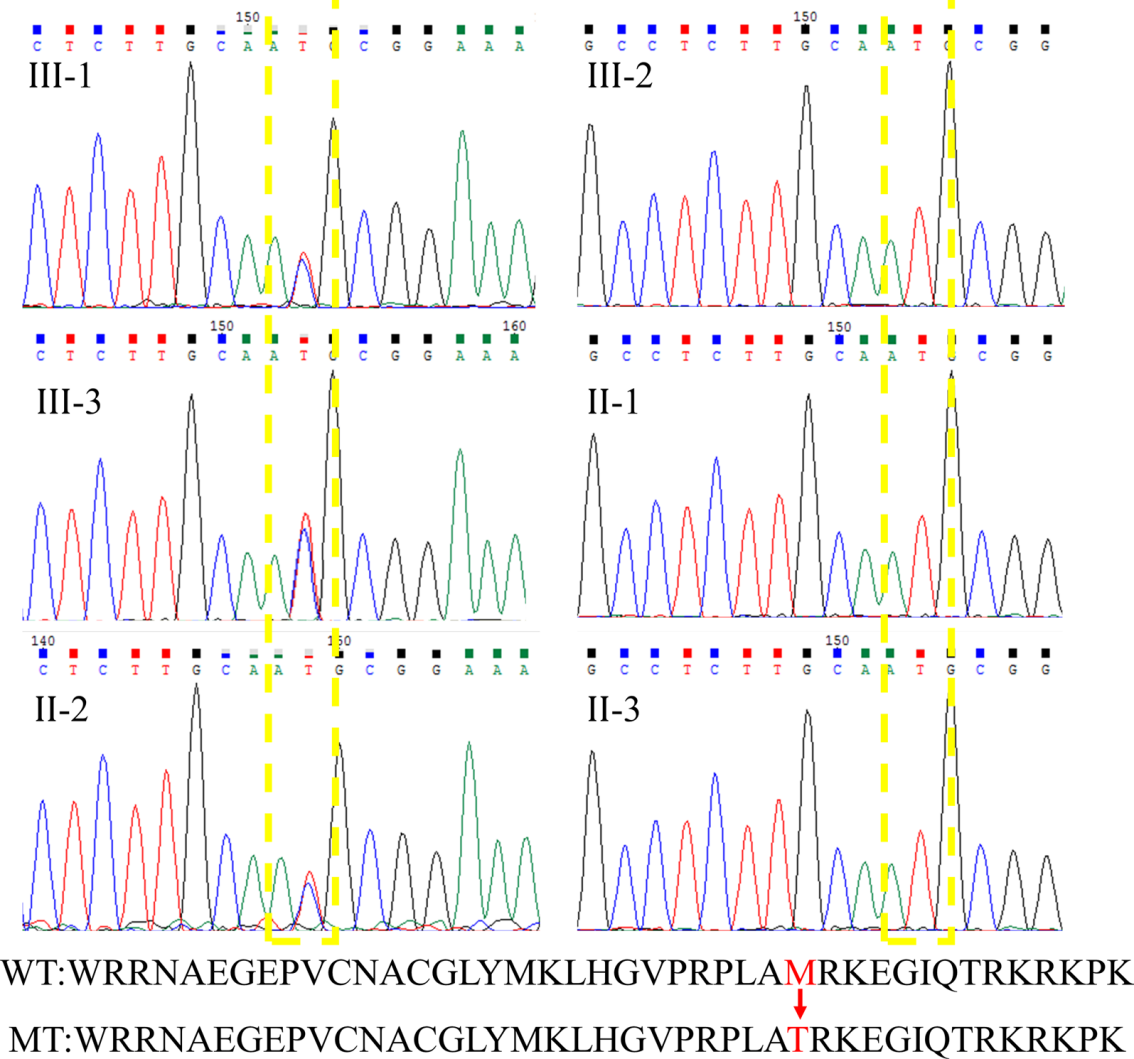
All members with the secundum atrial septal defect had a *GATA4* mutation (left), a T-to-C transition at nucleotide 929 in exon 5 of *GATA4*. Other unaffected family members with wild type alleles of *GATA4* are shown (right); Wild type (WT) and mutated (MT) amino acid sequences of *GATA4* protein (bottom). The T929C transition creates a methionine to threonine substitution at codon 310

**Fig. 4:**
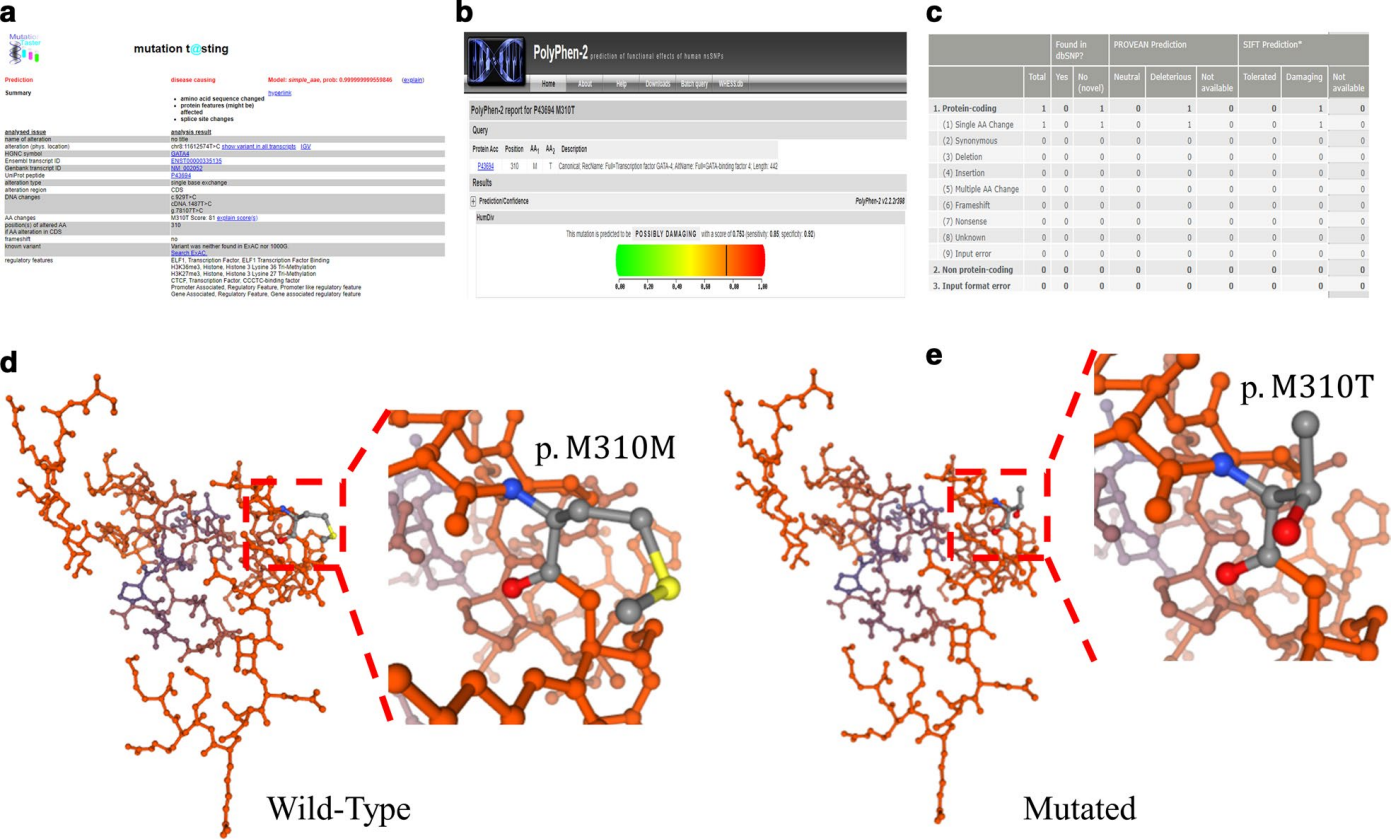
Results of several bioinformatics programs for the pathogenicity of the mutation of M310T. Results of Mutation Taster (**a**), Polymorphism Phenotyping v2 predictions (**b**), and Sorting Intolerant From Tolerant (**c**); The protein structure was determined using the Swiss Model tools (**d**, **e**)

M310T mutation was confirmed by Sanger sequencing from the samples of available members of the affected family (Fig. 3). At the same time, several bioinformatics programs (SIFT, Polyphen-2, Mutation Taster, PROVEAN, and LRT) were used to confirm the pathogenicity of the mutation of M310T, including the physical and chemical characteristics of amino acids at mutation sites, sequence conservation of mutation sites and their adjacent regions, protein structure characteristics, evolution characteristics, etc. The *GATA4* (c.T929C, p.M310T) gene mutation was predicted to be ‘Disease-causing’, ‘possibly damaging’, ‘Damaging’, ‘Deleterious’, and ‘Deleterious’ by Mutation Taster (Fig. 4a), PolyPhen-2 (Fig. 4b), SIFT (Fig. 4c) and LRT, respectively. In addition, compared with the wild-type (Fig. 4d), the potential effect of the mutant (Fig. 4e) on the protein structure was determined using the Swiss Model tools. Finally, we submitted the novel single nucleotide polymorphisms (SNPs) to the dbSNP database and received a submitted SNP (ss) number (2,137,544,112).

## Discussion

ASD is the third most common type of congenital heart disease, of which about 65–70% are secundum defects [25]. In these patients, changes in cardiac structure are triggered by increased blood flow resulting from a left-to-right shunt due to intracardiac defect. Therefore, early detection and surgical treatment are the main strategies. Thanks to the development of technique, including the introduction of cardiopulmonary bypass and accompanying large-scale heart surgery skills improvement, the mortality of ASD has been dramatically reduced [26]. However, due to the complexity of heart development, the exact causes of ASD, especially for the complex overlapping phenotype of ASD, still need more ongoing research efforts though we know that knowledge of heart development and function is the absolute necessity for better survival of human. Nowadays, the discovery of genetic causes of ASD has been being accelerated by many new technologies including single nucleotide polymorphism arrays, next-generation sequencing (NGS), and copy number variant platforms [27]. Among all new genetic technologies, the application of NGS in various aspects of heart biology has resulted in discoveries, generating novel insights into this field of study [28]. In the present study, WES combined ROH was employed to find out the suspicious disease-causing gene in one consanguineous family. This technique has been considered as a rapid and cost-effective tool for screening the new variants or genes for rare Mendelian unknown disorders [29, 30]. It helps genetic diagnostics for clinical cases with a mutational spectrum of known and unknown diseases. Some filtering strategies are needed for excluding variants that are implausible to cause disease because sometimes it is difficult to identify between pathogenic and benign mutations in the WES results [31]. With the analysis of WES result in this study, we quickly determined the most possible pathogenic mutation in this family is *GATA4* p.M310T and confirmed the result by Sanger sequencing.

*GATA4* gene belongs to a GATA family, which is consisted of 6 structure-conserved transcription factors. *GATA4* gene, which is expressed in the cardiac system and endodermal derivatives [32], is a highly conserved transcriptional factor with seven exons. The *GATA4* protein is comprised of TAD, NZF and NLS [2, 33]. Many shreds of evidence showed that *GATA4* plays significant roles in many stages of heart development, including looping morphogenesis, septation, ventricular myocardium proliferation, and heart contraction [34]. For this reason, *GATA4* was considered as a regular candidate for CHD genetic screening. Many mutations in the *GATA4* coding region have been identified as the genotype of CHD patients and not all of them were predicted by bioinformatics tools, such as PROVEAN and SIFT, as the pathogenic genes [34]. NLS was considered as a crucial role in ASD epidemiology because 5 family cases were reported in this region, include S52F, G296S, 1074 (delC), 1075 (delG) and M310V [2, 3, 35]. Especially in our present study, we determined a different protein mutation in the same protein location (M310V) as reported [2]. What’s more, in the mouse mutation study, M310V transgenic mice had shown a higher incidence of CHD than wild-type control mice [36], which indicated codon 310 in the *GATA4* gene is a CHD-related pathogenic coding region.

Here, we report for the first time an M310T mutation in the NLS region, which is necessary and sufficient for *GATA4* transcription factor activity and cardiac development. Using the Swiss model tool, we identified the NLS region as the potential impact region of the mutation on protein structure (Fig. 4). The changes in this region may lead to a decrease of transcriptional activity, thus affecting the transcriptional activation process during development. What's more, the region affected by M310T mutation is also immediately adjacent to CZf region, which is crucial for DNA binding and cofactor interaction [36, 37]. In addition, Garg reported [14] that the G296S mutation disrupts the DNA-binding and transactivation activity of *GATA4* and destroys the synergy in transcriptional activation between *GATA4* and its cofactor *TBX5*, resulting in heart anomalies such as pulmonary stenosis, atrioventricular septal defect, and ASD. Therefore, *GATA4* mutation in NLS region may also affect the expression of other transcription factors (*TBX5* or *NKX2.5*) [14, 35], which are crucial in the development of heart, resulting in the observation of ASD in three affected members of this family.

ASD families mostly present the same subtype structural defects without arrhythmia. In this study, all affected family members presented similar defects with distinct differences and all 3 patients were detected different tachyarrhythmia. Although some reported arrhythmias and conduction disorders may be associated with atrial septal defects [38], the reason is unknown and some transcriptional factors genes mutations were related to the ASD family with arrhythmias [39], one possible evidence shown by computationally Mattapally et al. [40], where they established that *NKX2.5* cooperativity with *GATA4* facilitates its activating and repressing functions [41–43]. The interaction between *NKX2.5* (TN domain) and *GATA4* might also be important for the function as a repressor of ion channels and its downstream target genes. Therefore, they speculate that mutation present in TN domain of *NKX2.5* gene will result loss of *NKX2.5* and *GATA4* interaction, thus will lead to loss of several activator and repressor function of this complex. *GATA4* p.M310T in future studies we also need show any effect activator and repressor function have trachyarrythemia. Further investigation is needed for the different phenotypes with the same genotype in the ASD family with arrhythmias.

CHD is a heavy load for the young family and the whole society. In China, the prevalence is still high although many CHD fetuses were aborted when the prenatal screening predicted highly possible of CHD. In the past two decades, cardiac development and genetic studies had provided much detailed information and identified many critical genes in the development of the heart. Although many mutants of these genes had been screened out, more biochemical methods and vivo models should be adopted for confirming. A limitation of this study is that only genetic screening methods had been employed but no furthermore study, such as vivo models, were used. Further studies will be conducted in the future to study the pathogenic mechanism of ASD families with arrhythmias and the reason for different phenotypes with the same genotype.

## Conclusion

We identified for the first time a novel M310T mutation in the *GATA4* gene that is located in the NLS region and leads to family ASD with arrhythmias. However, the mechanism by which this pathogenic mutation contributes to the development of heart defect and tachyarrhythmia remains to be ascertained.

## Supplementary information

**Additional file 1:** The detailed information of whole-exome sequencing data.

## Data Availability

The datasets generated and/or analyzed during the current study are available in the dbSNP database repository, persistent web link: https://www.ncbi.nlm.nih.gov/projects/SNP/snp_ss.cgi?subsnp_id=ss2137544112.

## Abbreviations

ASD: Atrial septal defect
CHD: Congenital heart defects
PVS: Pulmonary valve stenosis
VSD: Ventricular septal defect
PAVSD: Partial atrioventricular septal defect
TAD: Transactivation domains
NZf: N-terminus zinc fingers
CZf: C-terminus zinc fingers
NLS: Nuclear localization signal
WES: Whole-exome sequencing
HTS: High-throughput sequencing
SNVs: Single nucleotide variants
ROH: Runs of homozygosity
SNPs: Single nucleotide polymorphisms
