# Supplementary material for: The M310T mutation in the GATA4 gene is a novel pathogenic target of the familial atrial septal defect
Source: BMC Cardiovasc Disord. 2021 Jan 6;21:12. doi: 10.1186/s12872-020-01822-5 (PMC7788758; doi:10.1186/s12872-020-01822-5)
Supplement: Supplementary file 1 — Additional file 1: The detailed information of whole-exome sequencing data. [file 12872_2020_1822_MOESM1_ESM.docx]

| **Sample** | **Number** |
| --- | --- |
| Target Region (bp) | 51,542,852 |
| Clean Reads | 31,575,947 |
| Clean Bases (Mb) | 4,724.73 |
| Reads Mapped to Genome | 31,518,594 |
| Map Rate (%) | 99.82 |
| Reads Mapped to Target Region | 22,108,564 |
| Capture Specificity (%) | 70.14 |
| Duplication Rate (%) | 4.86 |
| Uniq Rate (%) | 95.47 |
| Uniq Reads Mapped to Target Region | 20,257,110 |
| Mean Depth of Target Region | 47.33 |
| Coverage of Target Region (%) | 95.71 |
| Rate of Nucleotide Mismatch (%) | 0.36 |
| Fraction of Target Covered ≥ 4X | 92.73 |
| Fraction of Target Covered ≥ 10X | 87.83 |
| Fraction of Target Covered ≥ 20X | 77.75 |
| Fraction of Target Covered ≥ 30X | 65.48 |
| Fraction of Target Covered ≥ 40X | 53.01 |
| Uniq Reads Mapped to Flanking Region | 12,199,351 |
| Mean Depth of Flanking Region | 16.3 |
| Coverage of Flanking Region (%) | 87.88 |
| Fraction of Flanking Covered ≥ 4X | 71.22 |

Additional file 1: Table S1: The detail information of whole-exome sequencing
